# Investigating the Relative Risk Factors of Injuries Caused by Accidents on Roads in the Mashhad Area in 2007

**Published:** 2011-08-01

**Authors:** A Vafaee-Najar, M Khabbazkhoob, H Alidadi-Soltangholi, S Asgari, H Ibrahimipour

**Affiliations:** 1 Health Sciences Research Center, School of Public Health, Mashhad University of Medical Sciences, Mashhad, Iran; 2 Department of Health and Management, School of Public Health, Mashhad University of Medical Sciences, Mashhad, Iran; 3 Noor Ophthalmology Research Center, Noor Eye Hospital, Tehran, Iran; 4 Department of Environmental Health, School of Public Health, Mashhad University of Medical Sciences, Mashhad, Iran

**Keywords:** Accidents, Case-control study, Iran

## Abstract

**Background:**

Currently, accidents are the second highest cause of death in most societies. Traffic accidents account for the largest proportion of accidental deaths. The aim of this report was to identify the accidents that cause casualties on the roads around Mashhad.

**Methods:**

This study was a case-control study, where the cases were drivers who had accidents resulted in casualties, and the controls were drivers who had accidents in the same locations without casualties. Variables included age, sex, seatbelt use, spontaneous combustion, entrapment within the vehicle, ejection from the vehicle, music playing in the vehicle at the time of the accident, use of cell phone, smoking at the time of the accident, the direction of the accident, the time of day, and the model of the vehicle.

**Results:**

Interviews were conducted with the 90% of the cases and the 93% of the controls who consented to being interviewed. Females accounted for 16.2% of the case group and 23.4% of the control group, and males comprised 83.8 % of the cases and 76.6% of the controls. The average age of the case group was 35.5±10.5 and of the control group was 39.4±9.8 years. The use of a seatbelt as a safety factor was significantly greater in the control group (OR=0.44). Combustion occurred in approximately 21% of the accidents in the case group, but in only 1.3% of the accidents in the control group. Being trapped in and being ejected from the vehicle were significantly more prevalent in the case group.

**Conclusions:**

According to the results of this study, the fastening of seatbelts had a significantly positive effect on reducing the injuries caused by an accident. Age was another significant indicator influencing the outcome of road accidents education through media seems to play a great role in reducing mortality and morbidity due to road accident.

## Introduction

According to the report by World Health Organization (WHO), an accident is defined as an incident with no history of causing a recognizable harm. Based on WHO annual statistics, approximately 1.2 million deaths were caused by accidents each year. Some other statistics reported 226 deaths out of 1 million in year 2000.

It is expected that until year 2010, deaths caused by accidents reach to 1.8 million per year. It is predicted that in year 2010, the load caused by accidents reaches the 3rd rank.[1][2] The reported results from our country indicate a higher number in comparison to the Eastern Mediterranean region and some studies reported the frequency of 30 in 100 thousands.[3] According to a report on 2005, over 25 thousands death in car accidents were reported to the legal medical centers, this rate is 17% more than its equivalent in the previous year. It is also estimated that every hour, 3 deaths occur due to car accidents.[4] Car accident besides the damages they cause, have consequences like injuries and disabilities which are intolerable for the societies. In our country, the number of car accident inpatients in year 2000 was reported 88.4 in 10000.[3] Besides the harm and damages that accidents have for the individuals, there might be as well injuries and disabilities which can affect the future life of the individuals and reduce the quality of life. Accidents as well may have consequences in the economical life of the individuals.

Most accidents cause unpredicted costs for the societies while compensating these costs especially for the developing countries are difficult. In our country, the cost of car accidents is about 1 billion Dollars per

year.[3] This study aims at investigating the risk factors in the road accidents injuries around Mahshad City, Western Iran in 2007.

## Materials and Methods

In a case-control study conducted from June to November 2007, the cases were the drivers who drove on these roads and had a history of accident and the control group was the drivers driving in the same roads, having accidents but experiencing no damages or injuries. Most of the enquiries were performed at the place of accident or the emergency centers near the scenes.

In the control group, the interview was performed as much as possible with the drivers and in the case group depending on the condition of the injured and level of consciousness, the interview was done with the drivers or ones accompanying him or her, in some cases the interview was postponed to the time that the injured driver became conscious later. The instrument for enquiry was questionnaire, the reliability and validity of which were tested beforehand, the interviewers had at least high school diploma and were trained to minimize the measurement errors at the time of investigation as much as possible.

The effect of 12 independent variables was investigated on the accidents. Variables under investigation were age, sex, seat belt, spontaneous combustion at the accident, trapping at the accident, falling out of the vehicle at the accident time, music playing in the vehicle at the time of the accident, using cell phone, the direction of the accident, the time, smoking at the time of the accident, and the model of the vehicle in both groups.

The sampling was done based on the type of the study and to investigate the effect of use or not using a seat belt at the time of accident among the control and case groups. The sampling was based on the findings of the previous studies in which the probability was reported to be 1.53, with an error of 5% and power of 80%. In the case and control groups, 170 samples were enrolled.

Based on the type of the study and the small sample size, the sampling was done based on the objective-based sampling procedure and the sampling continued until the sampling population was achieved. In this study, all samples were selected from the Asian roads directing to Mashhad. The roads were Mashhad-Nayshabur, Mashhad-Torbat Heidariyeh, Mahshad- Taybaad and Mashhad-Ghuchan.

It is worth mentioning that the design of this study exactly follows the design that Khalaji et al. used in their previous study.5 Our questionnaire and questions were also similar. The objective behind using the same instruments as khalaji et al. was to investigate the effect of the variables under study, applying the same methodology as well helping the authors to draw conclusions by means of comparing these studies.

The data were analyzed by SPSS (Version 15, Chicago, IL, USA) and STATA software based on the objective of this study to investigate the risk factors related to the drivers’ condition in the roads around Mashhad using logistic regression model. After indicating the risk factors in different logistic regression models, in order to find the ultimate risk factors and separating them from the interrupters, a backward logistic regression was applied.

In this model, in a multi-model based on the highest non-significant p-value, the variables were omitted from the model and the ultimate model was based on the p-value less than 0.05 in a multi-model consisting of all remaining variables.

## Results

Among the people who had the required characteristics of the study, 90% in the case group and 93% of the control group were willing or able to be interviewed. As Table 1 indicates, approximately 80% of the subjects (250 persons), were male and the 62 remaining persons were female.

**Table 1:** Distribution of case and control by sex and age

**Age (year)**	** Case **		** Control**		**Total**
	** Female **	** Male **	** Female **	** Male **	
	**No. (%)**	**No. (%)**	**No. (%)**	**No. (%)**	
< 20	0	3 (2.5)	2 (8)	5 (3.9)	10 (3.2)
20–29	4 (10.8)	20 (16.5)	8 (32)	45 (34.9)	77 (24.7)
30–39	32 (59.9)	49 (40.5)	1 (4)	45 (34.9)	117 (37.5)
40–49	9 (24.3)	30 (24.8)	14 (56)	22 (17.1)	24 (75)
50–59	2 (5.4)	16 (13.2)	0	10 (7.8)	28 (9)
> 60	0	3 (2.5)	0	2 (1.6)	5 (1.6)
	37 (100)	121 (100)	25 (100)	129 (100)	312 (100)

The average age of the participants in this study was 37.4±10.3 years. The age range of the participants was 18 to 69 years old and 16.2% of the case group and 23% of the control group were female. The distribution of males in both case and control groups was 83.8% and 76.6% respectively. Regression did not indicate any significant relationship between the risk and injuries with sex in the case and control group (p>0.05). The mean of the case group was 35.3±10.5 and in the control group was 39.4±9.8.

The average age of the participants in the case group and control group was statistically different (p<0.001). As is indicated in Table 2, 42.9% of the case group used seat belts at the time of the accident but this amount was 62.7% in the control group. This difference was statistically significant (OR=0.44) in a single variable model. To rephrase, it can be asserted that the chance of getting injuries in the case of not using seat belts in comparison to using seat belts was 2.5 times more.

**Table 2:** Distribution of case and control by risk factors

**Risk factor **		**Case**	**Control**
		** No. (%)**	**No. (%)**
Sex	Male	129 (83.8)	121 (76.6)
	Female	25 (16.2)	37 (23.4)
Fastening seatbelt	No	88 (57.1)	59 (37.3)
	Yes	66 (42.9)	99 (62.7)
Trapped within the car	No	107 (69.5)	133 (84.2)
	Yes	47 (30.5)	25 (15.8)
Ejection from the car	No	109 (70.8)	149 (94.3)
	Yes	45 (29.2)	9 (5.7)
Car Model	No	69 (44.8)	77 (48.7)
	Yes	85 (55.2)	81 (51.3)
Combustion	No	121 (79.1)	156 (98.7)
	Yes	32 (20.9)	2 (1.3)
Listening to music	No	76 (49.4)	86 (54.4)
	Yes	78 (50.6)	72 (45.6)
Smoking	No	96 (62.7)	136 (86.1)
	Yes	57 (37.3)	22 (13.9)
Using handheld phone	No	131 (85.1)	137 (80.4)
	Yes	23 (14.9)	31 (19.6)
Time of accident	8:00–12:00	63 (40.9)	64 (40.5)
	12:00–18:00	26 (16.9)	59 (37.3)
	18:00–24:00	65 (42.2)	35 (22.2)
Direction of contact	Behind	38 (24.7)	67 (42.4)
	Front	84 (54.5)	48 (30.4)
	Next	32 (20.8)	43 (27.2)
Total		154 (100)	158 (100)

The relationship between sex and not using seat belts in getting injuries was statistically significant in males (p<0.001). In Table 3. the related results to injuries and investigated variables were indicated in single and multi-variable logistic regression model. It is worth mentioning that fastening seat belt was approximately 5 times more in women, of course the relationship between not fastening seat belt and getting injuries can not be due to the interfering effect of sex, since there was no significant relationship between this event and sex. As was shown in Tables 1, 2 and 3, the model of the car did not have any statistical significance in the injuries (p>0.05).

**Table 3:** Association of physical accident with risk factors by logistic regression

**Variable**		**OR a**	**95% CI **	**P value**
Age	Year	0.96	0.93-0.98	< 0.001
Sex	Female	1		0.114
	Male	1.57	0.9-2.77	
Fastening seatbelt	Yes	1		< 0.001
	No	2.23	1.42-3.52	
Ejection from the car	No	1		< 0.001
	Yes	6.83	3.2-14.52	
Trapped within the car	No	1		0.002
	Yes	2.33	1.35-4.04	
Listening to music	No	1		0.369
	Yes	1.22	078-1.91	
Car Model	No	1		0.487
	Yes	1.17	0.75-1.82	
Combustion	No	1		< 0.001
	Yes	20.6	4.84-87.8	
Smoking	No	1		0.046
	Yes	1.81	0.39-1.03	
Using handheld phone	No	1		< 0.275
	Yes	0.71	0.39-1.3	
Time of accident	8-12	1		0.006
	12-18	0.44	0.25-0.79	
	18-24	1.81	1.1-3.2	0.021
Direction of contact	Behind	1		< 0.001
	Front	3.08	1.81-5.25	
	Next	1.31	0.71-2.40	0.38

^a^ Adjusted odds ratio

Combustion in the case group was approximately 21% which was 1.3% in the control group, indicating that the chance of combustion in the case group was 20 times more than the control group in a single variable analysis. This variable in comparison to the other influencing variables significantly was correlated with the degree of injuries and risks caused by the accidents. The results indicated that 30.5% of the cases were trapped at the time of the accident; this number in the control group was approximately 15.8%. This model both in the single and multiple model were significantly correlated with the amount of injuries and its risk (p<0.002). Of course, this variable along with the model of the car had a weaker correlation in the case and control group which was again significant after the omission of the model of the car. Trapping in the car in both case and control groups was significantly higher in the less modern cars, (p<0.05). Based on this variable as well, falling out of the car was significantly different in two groups (p<0.001). The relationship indicated in the multiple variable groups beside not using seat belts, it was significantly higher and after precise investigations indicated that only 4.2% of the people with seat belts were fallen out of the car during the accident, mentioning that this number was 32% among the ones not fastening seat belts. Results indicated that the chance of falling out of the car during the accident in case of not using seat belts was 10.2 times more (p<0.001). Smoking was also significantly correlated in the case group with the time of accident (p<0.05). In the multiple-variable model, this variable along with the sex indicated a non- significant correlation, other variables such as listening to music or talking with a hand phone did not have a significant correlation with the extent of injuries.

After entering all variables into the multiple logistic regression model, 3 variables, include age, not fastening seat belt and combustion, were identified as more dangerous risk factors. Comparing Table 3 and this finding indicates that smoking and the time and direction of the accident were among the variables which were shown to have a significant correlation in the single variable model, but were not considered significant in the presence of other variables. Of course, it is worth mentioning that in a basic multivariable model, falling out of the car and being trapped at the time of accident were remained in the model and were correlated with the amount and severity of the injuries. But since falling out of the car was significantly higher in cases not using seat belts, this variable was omitted from the model and the trapping was omitted with it as well.

## Discussion

This study is a case-Control study which aims at investigating the danger factors of accidents occurring in the roads around Mashhad, in year 2007. The selection of the control in case-control studies is always considered as an important variable, in this study although the control group was selected from the drivers having accidents in the same roads but due to some demographic variables, there were some measurement errors which were considered as the limitations of this study, which may influence the accuracy of the study too. Another factor in this study was the critical conditions of the case group which made them unable or unwilling to be interviewed; therefore some severe cases were automatically omitted from our study. But still since we were able to study at least 90% of the cases in both groups, this error was minimized. Another limitation of our study was that we could not get honest and clear answers about the possibility of using drugs, which may act as interference and affect some relations in positive or negative way. Due to the mentioned limitations and small number of population, care must be given while comparing the results of the study with other cases. The severity of the accidents is also considered as an important factor, the studies in this regard indicate that this factor may influence other variables. This factor is highly correlated with pace and weight of the vehicle, even though it is important to remember that we have not focused on the severity of the accidents, and it may only be considered as an interrupting variable in our study.[5] For instance, injures, combustion and trapping and some other variables may correlate with the severity effect, therefore it is important to consider this variable.

In order to facilitate the comparison, our results have been presented with Khalaji et al.’s results in Table 4.[5] Several studies have been conducted in this regard all around the world, but due to differences in life style, roads condition, and other related factors the comparison may not be considered as accurate.

**Table 4:** Comparing this study with Khalaji et al.’s study.

**Variable**		**Our study**		**Khalaji et al.’s study**	
		**Case %**	**Contorol %**	**Case %**	**Contorol %**
Fastening seat belt		9.42	62.7	42.8	57.2
Being thrown out of the car		29.2	5.7	26.9	5.1
Car Model		55.2	51.3	53.1	50.9
Combustion		20.9	1.3	6.3	0
Direction of Contact	Behind	24.7	42.4	12	16
	Front	54.5	30.4	46.3	36
	Next	20.8	27.2	41.2	47.9

Among the studies conducted so far, khalaji et al.’s study which considered Qazvin-Lushan road in year 2005 with the same methodology seems to be a profound and proper case for comparison.[5] It is also worth mentioning that most studies in this regard in our country have been descriptive, so testing hypothesis with analytical studies may contribute a lot to the existing knowledge and conclusions.[6][7][8][9][10][11][12][13]

It seems that sex can be deemed as an important variable to begin comparison within such studies, as this study also indicated that sex did not have a significant effect on the accidents in both case and control groups.

Although the amount of accidents was higher in men,[12] but most studies indicated that, the severity of accidents was similar in both groups. [5][14] Some studies have indicated the more severity of accidents in men.[15] The average age of the case group is reported 5 years less than the control group; most studies indicate that lower age of driver were correlated with the severity of the accidents. [15] Of course, some other studies such as Khalaji et al. and Norris et al. have rejected the correlation of the age factor with the severity of the accidents.[5][14] Khalaji et al.’s study indicates that 42.8% of the case and 57.2% of the control group used protective tools such as seat belt.5 In the present study, the fastening of seat belt in the case group was reported as 42.9% and in control group 62.7%, this shows that our result were correlated with khalaji et al.’s results supporting the positive role of seat belt in reducing accident’s severity.[5] The importance of using seat belt is now obvious and has been proved in various studies , of course it is worth mentioning that using seat belt can decrease the severity of the accidents and injuries caused by them, but has no effect in preventing the occurrence of the accidents.[1][5][9][13][16][17]

A study conducted in year 2006 indicated that two third of the death injuries in car accidents were related to passengers and drivers not fastening seat belts.18 The time of the accident is considered as another important factor to be studied, it is expected that due to the decrease of eye sight during the late mid-night the number of accidents increases, but our results rejected this assumption. In other studies, it was shown that 72% of the accidents occurred from 6 am to 18 pm, of course some results from the traffic police indicated that most accidents occurred during 5 to 8 am and 16 to 20 pm.[4][7][13]

Of course, these results can not be generalized since they have been descriptive and did not investigate the effect of the time of the accidents on the severity of the accidents. The present study shows the correlation of the time of the accident with the severity of the accidents and as it is indicated in the results, the severity of the accidents during 12-18 pm has been less than 6-12 am, the accidents occurring during 18-24 pm as well have been more severe in comparison to the accidents taking place during 6-12 am.

We have indicated in this study that 20.9% of the case group experienced combustion during the accident, but this number is reported to be 1.3% in the control group. The influence of this factor on the other hand is important on the injuries to the drivers and passengers which are both due to the severe contact and the fire. In khalaji et al.’s study, this number is reported smaller than the present study i.e. 6.3% in the case group and 0% in the control group.[5]

The modernity or model factor of the cars during accident was also studied in both researches, although no significant difference was observed in both groups but generally the number of modern cars was higher in the case group which may relate to the higher speed of these cars or the relaxation effect caused by the comfort of the more modern cars which can make the driver less cautious while driving on the road and consequently cause accident. We can conclude that using seat belt can decrease the severity of the injuries caused by the accidents or prevent the injury of the driver. The low age of the driver may also be considered as a risk factor which can cause accidents on the roads and can be minimized by means of media awareness and training.
